# Blood Glucose and Lactate Levels and Cerebral Oxygenation in Preterm and Term Neonates—A Systematic Qualitative Review of the Literature

**DOI:** 10.3389/fped.2020.00361

**Published:** 2020-07-29

**Authors:** Christian Mattersberger, Georg M. Schmölzer, Berndt Urlesberger, Gerhard Pichler

**Affiliations:** ^1^Division of Neonatology, Department of Paediatrics, Medical University of Graz, Graz, Austria; ^2^Research Unit for Neonatal Micro- and Macrocirculation, Department of Paediatrics, Medical University of Graz, Graz, Austria; ^3^Centre for the Studies of Asphyxia and Resuscitation, Royal Alexandra Hospital, Edmonton, AB, Canada; ^4^Department of Pediatrics, University of Alberta, Edmonton, AB, Canada

**Keywords:** neonates, blood glucose, lactate, near-infrared spectroscopy, cerebral oxygenation

## Abstract

**Background:** Cerebral oxygenation monitored non-invasively by near-infrared spectroscopy (NIRS) is of increasing interest in neonatal care. Cerebral oxygenation is determined by cerebral oxygen delivery and cerebral oxygen consumption. Oxygen delivery as well as oxygen consumption might be influenced by metabolic parameters like blood glucose and lactate.

**Objective:** The aim of the present systematic qualitative review is therefore to identify and summarize all studies, which describe cerebral oxygenation measured with NIRS and blood glucose and/or blood lactate levels in neonates.

**Data sources:** A systematic search of Ovid Embase and PubMed was performed. Search terms included near-infrared spectroscopy, fractional tissue oxygen extraction, cerebral tissue oxygen saturation, regional cerebral tissue oxygen saturation, oxygenation, term, and preterm neonates, cesarean delivery, transition, after-birth, newborn, vaginal delivery, cesarean delivery, baby, neonatal transition, metabolism, lactate, glucose, and blood glucose level.

**Study selection/data synthesis:** Studies analyzing cerebral oxygenation and blood glucose and/or blood lactate levels in neonates were included. Animal studies, duplicates, or studies in non-English language were excluded.

**Results:** Twenty-five studies were identified that describe blood glucose and/or blood lactate levels as primary or secondary outcome parameters with additional measured cerebral oxygenation by NIRS in neonates. Twelve studies were included with blood glucose measurements: four described an association between blood glucose levels and cerebral oxygenation, two show no association, and six do not report on possible associations. Eighteen studies were included with lactate measurements: one describe an association between lactate levels and cerebral oxygenation, while three show no association and 14 do not report on possible associations.

**Discussion:** The influence of blood glucose and blood lactate levels on the cerebral oxygenation in neonates is still controversial. However, there seems to be an association between cerebral oxygenation and the metabolic parameter blood glucose and lactate, which need further investigation.

## Introduction

Irreversible cerebral injury due to impaired cerebral oxygenation is a persisting problem in the neonatal period despite improved monitoring and intervention options. Standard non-invasive monitoring in neonatal care does not yet assess cerebral oxygenation, oxygen delivery to the brain, or cerebral oxygen consumption (1–3). However, cerebral near-infrared spectroscopy (NIRS) monitoring has the potential to detect impaired cerebral oxygenation in neonates while other vital parameters such as arterial oxygen saturation or heart rate remain within their normal range (4). NIRS is a continuous, non-invasive monitoring technique to measure the cerebral oxygenation in neonates and measures the cerebral regional oxygen saturation and fractional tissue oxygen extraction. A recently published multicenter trial using cerebral NIRS monitoring to reduce the burden of cerebral hypoxia in preterm neonates described beside cardiovascular and respiratory interventions also interventions based on blood glucose levels (5). Another recently published study describe an association between blood glucose level and cerebral oxygenation in preterm and term neonates immediately after birth (6). Further, lactate as a product of anaerobic metabolism might be associated with hypoxic conditions in the tissue. An association between the blood lactate level and the cerebral oxygenation has been described in extremely preterm neonates during the 1st days after birth (7).

The aim of the present systematic qualitative review is therefore to identify and summarize all studies, which describe cerebral oxygenation measured with NIRS and blood glucose and/or blood lactate levels in neonates.

## Methods

### Search Strategy and Selection Criteria

Studies were identified using the stepwise approach specified in the Preferred Reporting Items for Systematic Reviews and Meta-Analysis (PRISMA) Statement (8).

### Eligibility Criteria

Studies had to address cerebral oxygenation measurements with NIRS as well as the metabolic parameters blood glucose and/or lactate in neonates.

### Search Strategy

A systematic search of Ovid Embase and PubMed NCBI was performed to identify studies in English language published between 1974 and November 2019. Search terms included near-infrared spectroscopy, fractional tissue oxygen extraction, cerebral tissue oxygen saturation, regional cerebral tissue oxygen saturation, oxygenation, term, and preterm neonates, cesarean delivery, neonatal transition, after-birth, newborns, vaginal delivery, baby, after cesarean delivery, metabolism, lactate, glucose, and blood glucose level.

### Inclusion and Exclusion Criteria—Population

To be eligible, studies had to investigate human neonates. Neonates were defined as infants with a postnatal age of <28 days. Studies that included neonates and infants or children were also included in our analysis, when the results were not separately analyzed for neonates. Animal studies were excluded.

### Inclusion and Exclusion Criteria—Measurements (Exposure)

We included studies with different NIRS devices, if any additional measurements of either capillary, venous, or arterial blood glucose levels and/or lactate levels were included.

### Inclusion and Exclusion Criteria—Types of Publication

We included clinical or observational studies published in English language. Non-original articles, such as comments, book chapters, editorials, reviews, and methods papers, were excluded. Duplications and publications in non-English languages were also excluded.

### Study Selection

The articles identified in the literature review were evaluated independently by two authors (CM and GP) for inclusion using the titles and abstracts. Then, full texts were retrieved and were included based on the eligibility criteria. Any disagreement was resolved through discussion and consensus between two authors. If there was uncertainty regarding eligibility for inclusion on the basis of the abstract, the full text was assessed too. Data were analyzed qualitatively. Data extraction included the study design, characterization of type (preterm/term) and number of neonates included in the study, applied device, NIRS and metabolic values, age of neonates during NIRS and metabolic measurements, and the presence or absence of any association.

### Risk of Bias in Individual Studies

A longer period between cerebral measurements and blood samples may originate a bias. Therefore, we included the exact time between cerebral measurements and blood samples in our qualitative analysis.

## Results

**Figure 1:**
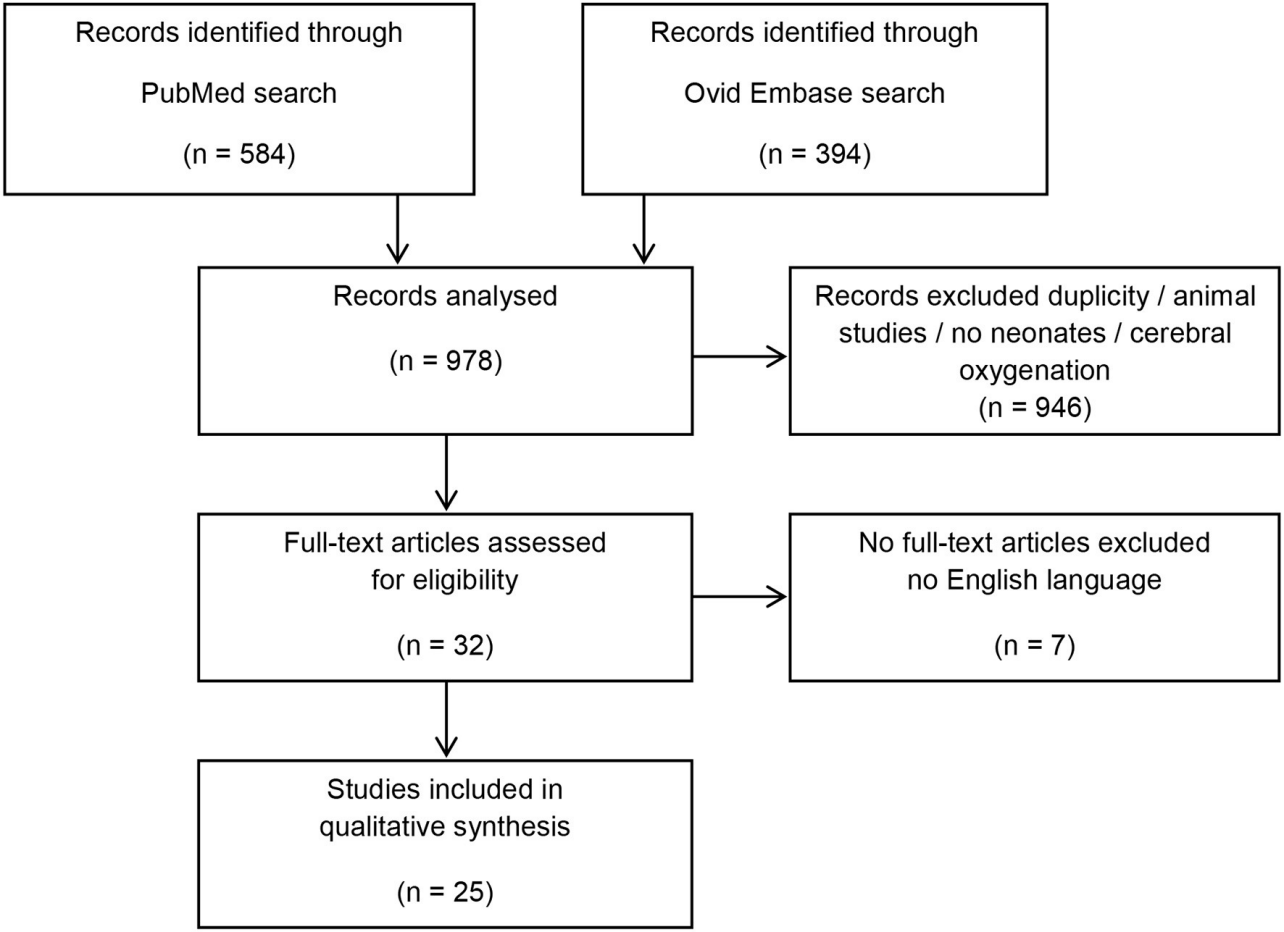
Flow diagram.

After the initial search, 978 abstracts were identified, which were assessed for eligibility. After full text search, 25 studies remained to be included in the present review (Figure 1) (6, 7, 9–31).

### Blood Glucose Level and Cerebral Oxygenation

Twelve studies were identified, which describe blood glucose level measurements in combination with cerebral NIRS measurements (Table 1) (6, 9–19). Four studies describe an association (6, 12, 14, 16) between blood glucose levels and cerebral oxygenation. All studies demonstrate a negative correlation. Two studies show no association (9, 11) and six studies do not report on possible associations (10, 13, 15, 17–19).

**Table 1:** Glucose and cerebral oxygenation in neonates.

**First author, Years**	**Study design**	**Neonates**	***n***	**Device**	**NIRS measurement,** **time point**	**Blood sample,** **time point**	**NIRS measurement,** **duration**	**TOI or crSO2**	**Blood-glucose-level,** **mean value**	**Association,** **correlation**
Naulaers G., 2002 (9)	Observational	Preterm	15	NIRO 300	Day 1–3 after birth	Before and after NIRS measurements	30 min	1 day 57% 2 day 66.1% 3 day 76.1%	n.r.	No
Naulaers G., 2003 (10)	Observational	Preterm	15	NIRO 300	Day 1–3 after birth	Before and after NIRS measurements	30 min	1 day 57% 2 day 66.1% 3 day 76.1%	n.r.	n.r.
Weiss M., 2005 (11)	Prospective observational	Preterm and term	155	NIRO 300	Day 12 (0–365) after birth	During NIRS measurements	30 min in 1 min intervals	60.5%	4.9 mmol/L	No
von Siebenthal K., 2005 (12)	Observational	Preterm	28	Critikon Cerebral Oxygenation Monitor 200	First 6 h after birth	n.r.	n.r.	n.r.	4.9 mM	Yes negativ
Bravo MDC., 2011 (13)	Prospective uncontrolled case series observational	Neonates and infants	16	NIRO 300	Day 5–70 after birth	Beginning and the end of the study	Continuously during 48 h in 20 s intervals	Δ −2.56%	n.r.	n.r.
Zhang G., 2012 (14)	Prospective observational	Neonates	17	INVOS 5100A	Day 7 (±4) after birth	2 to 4 h intervals	Continuously in 1 min intervals after surgery	n.r.	2.8-24.6 mmol/L	Yes negativ
Pellicer A., 2012 (15)	Pilot, phase 1 randomized, blinded clinical trail	Neonates	20	NIRO 300	Day 6–34 after birth	Before surgery, 6 h intervals during 24 h and 48 and 96 h	Immediately after surgery and continuously during the first day, for 4 h at 48 and 96 h postsurgery	n.r.	n.r.	n.r.
Li J., 2012 (16)	Observational	Neonates	17	INVOS 5100A	n.r.	n.r.	Continuously 72 h after surgery	n.r.	2.8–24.6mmol/L	Yes negativ
Weeke LC., 2017 (17)	Observational retrospective cohort	Preterm and term	25	INVOS 4100-5100	Preterm 120 h (46.5–441.4) term 20.7 h (7.2–131) after birth	4 h intervals	Continuously 10 min before, during and/or after hypercapnia	Before 66.54% during 68.36% after 65.91%	Before 6.64 mmol/L during 7.82 mmol/L after 6.96 mmol/L	n.r.
Nissen M., 2017 (18)	Retrospective observational	Preterm and term	12	INVOS 5100C	Day 43 (20-74) after birth	During NIRS, before restoration, before and after surgery	Before restoration of metabolic alkalosis, 3 h before, 16 and 24 h after surgery in 30 min intervals	Before restoration 72.74% before surgery 77.89% after surgery 80.79%	n.r.	n.r.
Mattersberger C., 2018 (6)	Observational	Preterm and term	75	INVOS 5100	Minute 15 after birth	Immediatly or up to 5 min after NIRS measurements	1 min	Preterm 80.2% term 83%	Preterm 2.7 mmol/L term 2.9 mmol/L	Yes negativ
Fister P., 2018 (19)	Observational case control	Term	65	INVOS 5100C	Case 15 days (10–20) controls 11 days (8–14) after birth	n.r.	5 min	Left 67 vs. 76% right 68 vs. 77%	Case 4.3 mmol/L controls 4.4 mmol/L	n.r.

*n.r., not reported; CHD, congenital heart disease; CPB, cardiopulmonary bypass; RCP, regional cerebral perfusion; NIRS, near-infrared spectroscopy*.

### Blood Lactate Level and Cerebral Oxygenation

Eighteen studies were identified, which describe blood lactate level measurements in combination with cerebral NIRS measurements (Table 2) (7, 11, 13, 15, 16, 18, 20–31). Only one study demonstrated a negative correlation between blood lactate levels and cerebral oxygenation (7). Three studies demonstrate no association (11, 23, 29) and 14 do not report on possible associations (13, 15, 16, 18, 20–22, 24–28, 30, 31). Five studies include blood glucose level as well as blood lactate level (11, 13, 15, 16, 18).

**Table 2A:** Lactate and cerebral oxygenation in neonates.

**First author, Years**	**Study design**	**Neonates**	***n***	**Device**	**NIRS measurement,** **time point**	**Blood sample,** **time point**	**NIRS measurement,** **duration**	**TOI or crSO2**	**Blood-lactate-level, mean value**	**Association, correlation**
Giacomuzzi C., 2005 (20)	Observational	Neonates	5	INVOS 5100B	day 17 (±18.9) after birth	Preoperatively, after initiation, on the first postoperative days of assistance	During surgery, cooling, circulatory arrest, rewarming, 24 and 48 h of assistance in 1 min intervals	Preoperatively 62.2% during cooling 80.2% during circulatory arrest 66.2% intermittent reperfusion 80.4% during rewarming 78.8% after bypass 42.8% 12 h assistance 48.2% 24 h assistance 57.2% 48 h assistance 60.6%	Preoperatively 1.98 during cooling 1.88 during circulatory arrest n.r. intermittent reperfusion 3.18 during rewarming 4.5 after bypass 4.6 12 h assistance 6.5 24 h assistance 1.68 48 h assistance 1.42	n.r.
Weiss M., 2005 (11)	Prospective observational	Preterm and term	155	NIRO 300	Day 12 (0–365) after birth	During NIRS measurements	30 min in 1 min intervals	60.5 %	2.6 mmol/L	No
Redlin M., 2008 (21)	Prospective observational	Neonates and infants	20	NIRO 200	Month 5.3 (±3.1) after birth	Simultaneously during NIRS measurements in 30 min intervals	Continuously before, during and after surgery and CPB	n.r.	n.r.	n.r.
Miyaji K., 2010 (22)	Prospective observational	Neonates and infants	18	INVOS 5100	Day 28 (±47) after birth	During the NIRS measurement at the beginning and end of the surgery, CPB, and RCP	Continuously in 1 min intervals at the beginning and end of the surgery, CPB, and RCP	Pre CPB 57.9% CPB cooling 68.6% RCP 78.8% CPB warming 66.8% post CPB 54.7%	Before 3.8 mmol/L after 5.5 mmol/L	n.r.
Bravo MDC., 2011 (13)	Prospective uncontrolled case series observational	Neonates and infants	16	NIRO 300	Day 5–42 after birth	Beginning and end of the study	Continuously during 48 h in 20 s intervals	Δ −2.56%	Initial 2.8 mmol/L final 1.7 mmol/L	n.r.
Amigoni A., 2011 (23)	Prospective observational	n.r.	16	INVOS 5100C	Month 3.5 (0–66) after birth	Before and after surgical procedure and at start, middle, and end of CPB	Continuously during surgical procedure	Basal 55% before CPB 42% CPB start 42.5% CPB middle 40.5% CPB before stop 41% CPB re-warming 46% after CPB 42.5% before discharge 50%	Basal 1.53 CPB start 1.85 CPB middle 1.98 CPB before stop 2.53 after CPB 3.25	No
Redlin M., 2011 (24)	Retrospective	Neonates	23	NIRO 200	Day 2–17 after birth	Pre- and postoperatively beginning, during and end of CPB	Continuously before and after surgery and CPB	Before surgery 90.7% and 89.9% start CPB 99.8% and 99.6% during CPB 99.7% and 99.5% end of CPB 99.7% and 99.0% after CPB 94.3% and 97.4% after surgery 62.7% and 59.5%	Before surgery 1.4 mmol/L and 1.3 mmol/L start CPB 2.0 mmol/L and 1.5 mmol/L during CPB 3.6 mmol/L and 2.4 mmol/L end of CPB 4.2 mmol/L and 2.4 mmol/L after CPB 4.0 mmol/L and 2.4 mmol/L	n.r.
Miyaji K., 2011 (25)	Retrospective	Neonates	17	INVOS 5100	Day 11.6 (±8.9) and day 12.5 (±15.6) after birth	During NIRS measurements	Surgical incision, initiation of CPB and RCP, at warming, end of CPB and surgery at 1 minutes intervals	83 and 66%	0.8 and 2.8 mmol/L	n.r.

*n.r., not reported; CHD, congenital heart disease; CPB, cardiopulmonary bypass; RCP, regional cerebral perfusion; NIRS, near-infrared spectroscopy*.

**Table 2B:** Lactate and Cerebral Oxygenation in Neonates.

**First author, Years**	**Study design**	**Neonates**	***n***	**Device**	**NIRS measurement, time point**	**Blood sample, time point**	**NIRS measurement, duration**	**TOI or crSO2**	**Blood-lactate-level, mean value**	**Association, correlation**
Pellicer A., 2012 (15)	Pilot, phase 1 randomized, blinded clinical trail	Neonates	20	NIRO 300	Day 6–34 after birth	Before surgery, 6 h intervals during first 24 h, and once at 48 and 96 h	Immediately after surgery and continuously throughout the 1st day, for 4 h at 48 and 96 h postsurgery	n.r.	n.r.	n.r.
Li J., 2012 (16)	Observational	Neonates	17	INVOS 5100A	n.r.	n.r.	Continuously 72 h after surgery	n.r.	n.r.	n.r.
Haydin S., 2013 (26)	Retrospective	Neonates and pediatrics	50	Somanetics 5100B	Month 7 (0.2–168) after birth	10 min intervals during NIRS measurements	Beginning of CBP, during cooling and end of cooling, rewarming, before weaning	Beginning of CBP 55.7% during cooling 60.6% end of cooling therapy 59.6% rewarming 58.1% before weaning 59.8%	Beginning of CBP 2.8 during cooling 3.0 end of cooling therapy 3.1 rewarming 3.2 before weaning 3.5	n.r.
Gupta P., 2014 (27)	Retrospective observational	Neonates	15	n.r.	Day 19 (12–22) after birth	Before extubation	6 h before and 6 h after extubation	Extubation failure 56.0% and 57.0% extubation success 61.0% and 63.0%	Extubation failure 1.6 and 1.3 extubation success 1.2 and 1.5	n.r.
Mintzer JP., 2015 (28)	Prospective observational	Preterm	12	INVOS 5100C	Day 3 (2–5) after birth	During NIRS measurements	Continuously 1 h prior and 2 h immediately following procedure	74%	Before 0.9 mmol/L after 0.9 mmol/L	n.r.
Mebius MJ., 2016 (29)	Retrospective	Preterm and term	56	INVOS 4100C and 5100C	Day 0–3 after birth	Daily	Continuously within the first 72 h after birth	1 day 58.5% 2 day 62.5% 3 day 61.5%	3.9	No
Aly SA., 2017 (30)	Prospective observational	n.r.	75	NIRO 200	Day 5 (4–8) after birth	During NIRS measurements on CPB, 60 min off CPB and 24 h after surgery	30 min before, continuously during and for 24 h after surgery	Preoperativ 55% 60 min off CPB 55 and 43% 24 h after surgery 57 and 42%	During CPB 5.3 mmol/L 60 min off CPB 6.0 mmol/L 24 h after surgery 6.6 mmol/L	n.r.
Nissen M., 2017 (18)	Retrospective observational	Preterm and term	12	INVOS 5100C	Day 43 (20–74) after birth	During NIRS measurements, once before restoration, before and after surgery	Before restoration of metabolic alkalosis, 3 h before, 16 and 24 h after surgery in 30 min intervals	Before restoration 72.74% before surgery 77.89% after surgery 80.79%	n.r.	n.r.
Neunhoeffer F., 2017 (31)	Prospective observational	Neonates and infants	15	O2C device	Day 5 (1–150) and day 37 (1–68) after birth	Before operation, half-hourly during operation, and after surgery	Continuously during surgery	Before 61.85 vs. 65.02% during 66.75 vs. 67.62% after 66.75 vs. 69.87%	Before 0.8 vs. 1.1 mmol/L during 0.9 vs. 1.65 mmol/L after 1.0 vs. 1.42 mmol/L	n.r.
Janaillac M., 2018 (7)	Prospective observational	Preterm	20	INVOS 5100	Day 0–3 after birth	During NIRS measurements every 6–8 h	Continuously for 72 h in 30 min intervals	6 h 69% 24 h 76% 48 h 71% 72 h 68%	6 h 2.44 (μMol/L) 24 h 2.33 (μMol/L) 48 h 2.29 (μMol/L) 72 h 2.92 (μMol/L)	Yes negative

*n.r., not reported; CHD, congenital heart disease; CPB, cardiopulmonary bypass; RCP, regional cerebral perfusion; NIRS, near-infrared spectroscopy*.

Tables 1 and 2A,B give an overview of the data of the included studies.

None of the studies reported on possible simultaneous associations between both metabolic parameters (glucose and lactate) and cerebral oxygenation.

## Discussion

In the last few years, interest into research of cerebral oxygenation and metabolic parameters during the neonatal period increased significantly. There are several studies describing results of possible or missing association between metabolic parameters and cerebral oxygenation measured with NIRS. These results are controversial.

### Blood Glucose Level and Cerebral Oxygenation

Hyperglycemia has been identified as a risk factor for adverse outcome in critically ill patients (14, 16). The findings of the 12 identified studies (6, 9–19). with cerebral oxygenation measured with NIRS and blood glucose measurements are conflicting. Most studies described a negative association between cerebral oxygenation and blood glucose level (6, 12, 14, 16) with a decrease of cerebral oxygenation with increasing blood glucose levels. However, two studies described no association (9, 11). Naulears et al. (9) described an increase of cerebral oxygenation from day 1 to 3 after birth in neonates with postmenstrual age of 28 weeks. In this cohort, the multiple regression analysis showed no correlation between tissue oxygenation index and glycemia. In the largest cohort of neonates described by Weiss et al. (11) no association between blood glucose and cerebral oxygenation was observed. Interestingly, there was a negative association of blood glucose level with cerebral oxygenation observed in neonates after a Norwood procedure (14). Jia et al. (16) described a negative association between hyperglycemia and oxygen delivery. Further, she described a positive association between hyperglycemia and oxygen extraction ratio in neonates 72 h after Norwood procedure. Mattersberger et al. (6) demonstrated that blood glucose levels have a negative correlation to the cerebral oxygen saturation and a positive correlation to the cerebral fractional tissue oxygen extraction in preterm and term neonates 15 min after birth. Cerebral hemoglobin concentration that influences cerebral oxygenation, measured with NIRS, was investigated by Von Siebental K in neonates in the first 6 h of life. (12) He described different parameters influencing the cerebral hemoglobin concentration of neonates, whereby blood glucose had a negative correlation with cerebral hemoglobin concentration. The changes in cerebral hemoglobin concentration are in accordance with the above-described negative association between cerebral oxygenation and blood glucose levels when taking into account an auto-regulatory mechanism to maintain glucose supply to the brain. With decreasing blood glucose levels, there might be an increase in cerebral hemoglobin volume/concentration by increase of cerebral blood flow due to vasodilatation. This causes an increase in oxygen delivery with increase in cerebral oxygenation in case of a consistent cerebral oxygen consumption.

### Lactate Level and Cerebral Oxygenation

High lactate levels might be associated with an adverse neurologic outcome and can be a predictor for short-term neonatal adverse outcomes with similar predictive value as the pH value (32). Since lactate is a product of anaerobic metabolism, an increased level of lactate might represent hypoxic conditions in the tissue. Therefore, the interest in lactate in relation to the cerebral oxygenation in the neonatal period increased in the last years. Eighteen studies were identified, which investigated cerebral oxygenation and blood lactate level in neonates (7, 11, 13, 15, 16, 18, 20–31). However, only one of these publications demonstrated a negative association between cerebral oxygenation and lactate (7), and three studies found no association (11, 23, 29) between these factors.

Weiss et al. (11) described, in the largest cohort of critically ill neonates, no significant correlation between cerebral oxygenation and lactate. Amigoni et al. (23) also did not find an association between serum lactate and cerebral oxygenation. However, they described a correlation between pH value and cerebral oxygenation. Mebius et al. assessed the course of cerebral regional oxygen saturation and clinical factors in neonates born with duct-dependent congenital heart disease and found no correlation during the first 72 h after birth (29). In extremely preterm infants, it has been demonstrated that the crSO2 and preductal perfusion index were weakly correlated with lactate and blood gas (7).

## Limitation

The identified publications show many differences in methods: (e.g., study population, number of included neonates, NIRS devices, time point, and frequency of NIRS measurements). Important limitations are also the differences in frequencies of blood samples and differences in time periods between taking blood samples and NIRS measurements, ranging from 5 min (6) to 24 h (29). Several studies even provide no or inaccurate information on frequencies and time points of taking blood samples (11, 12, 16, 19, 27). This review identified only observational studies, where associations between cerebral oxygenation and blood glucose and/or lactate levels are described. No interventional study was identified elucidating any causality. Furthermore, there were several studies just describing cerebral oxygenation and blood glucose or lactate in neonates without analyzing any possible associations between these parameters.

## Conclusion

The influence of blood glucose level and blood lactate level on the cerebral oxygenation in neonates is still controversial. However, there is some evidence that there is an association between cerebral oxygenation and the metabolic parameters, blood glucose, and blood lactate, whereby causal relationship needs further investigation.

## Author Contributions

CM, GP, and BU: conception and design. CM and GP: literature search and drafting of the article. CM, GS, BU, and GP: analyses and interpretation of data, critical revision, editing, and final approval of the article. All authors contributed to the article and approved the submitted version.

## Conflict of Interest

The authors declare that the research was conducted in the absence of any commercial or financial relationships that could be construed as a potential conflict of interest.
